# Calretinin as a Diagnostic Adjunct for Ameloblastoma

**DOI:** 10.1155/2014/308240

**Published:** 2014-04-15

**Authors:** Chitra Anandani, Rashmi Metgud, Karanprakash Singh

**Affiliations:** ^1^Department of Oral and Maxillofacial Pathology, Pacific Dental College and Hospital, PAHER University, Udaipur, Rajasthan 313024, India; ^2^Department of Public Health Dentistry, Genesis Institute of Dental Sciences and Research, Ferozepur, Punjab 152001, India

## Abstract

*Background*. Calretinin is a 29 kDa calcium-binding protein of the EF-hand family which is expressed in a variety of normal and tumorigenic tissues. Its expression in odontogenic epithelium during odontogenesis and in neoplastic odontogenic tissues has been demonstrated. Unicystic ameloblastoma poses a diagnostic challenge, as its histologic presentation can be sometimes mistaken for keratocystic odontogenic tumor (KCOT). This study was performed to assess the usefulness of calretinin as a confirmatory marker for ameloblastic tissue. *Methodology*. Total of 40 cases: 16 unicystic ameloblastoma, 4 multicystic ameloblastoma, and 20 KCOT, were evaluated immunohistochemically for the presence, localization, distribution, and intensity of calretinin expression. Statistical analysis was done using Chi-square test to intercompare the expression between ameloblastoma and KCOT. *Results*. Sixteen cases of ameloblastoma (12 unicystic, 4 multicystic) showed positive calretinin staining of ameloblastic epithelium and only one case of KCOT was positive for calretinin, with the positivity restricted to the stellate reticulum like epithelium. Intercomparison between two groups revealed statistically significant difference (*P* = 0.000). *Conclusion*. Calretinin appears to be a specific immunohistochemical marker for neoplastic ameloblastic epithelium and may be an important diagnostic adjunct in the differential diagnosis of ameloblastoma and KCOT.

## 1. Introduction

Calcium ions (Ca^2+^) regulate a large number of biological processes such as metabolism, contraction, secretion, cell division, cell growth, and memory storage—either directly or indirectly. Elevated cytoplasmic calcium levels found in several tumor cells might contribute to the increased motility and hence invasiveness of these cells.

The calcium signal is transmitted into the intracellular response, in part via interactions with a wide variety of intracellular calcium-binding proteins that are involved in the regulation of many cellular activities. One class of these proteins shares a common calcium-binding motif, the EF-hand [1]. The EF-hand homologue family contains more than 200 different calcium-binding proteins, among them being 29 kDa calretinin, which was isolated initially from a cDNA clone from the chick retina, and it is most homologous to another important member of the EF-hand family, that is, 28 kDa calbindin [2].

Calretinin is widely expressed in central and peripheral neural tissues, particularly in the retina and in neurons of sensory pathways. Outside the central nervous system, it is expressed in normal and reactive mesothelial lining of all serosal membranes, the pilar infundibulum, eccrine glands, convoluted tubules of the kidney, leydig and sertoli cells of the testis, endometrium and ovarian stromal cells, adrenal cortex, epithelial cells of the thymus, and adipocytes [2, 3].

Studies in rats have demonstrated calretinin expression in neural elements of the tooth pulp, periodontal ligament and viscerosensory nerve fibres of oral and pharyngeal tissues [4], as well as in epithelium-derived tissues during odontogenesis in rat molar tooth germs, suggesting that this protein may play a part in enamel formation [2]. The exact biological function of calretinin remains unknown but its possible roles as a calcium buffer and/or calcium sensor and regulator of apoptosis have been postulated [5]. Calretinin also seems to share with calbindin certain features such as regulation of expression by growth factors and involvement in cell proliferation, differentiation, and neoplastic transformation [6].

Apart from its expression in normal human tissues, calretinin has been shown to be expressed in a wide variety of tumors [7]. Calretinin has recently emerged as an immunohistochemical marker with great utility for delineating mesothelioma and adenocarcinomas of lung, as its diagnostic sensitivity for mesothelioma is nearly 100% [8].

Odontogenic tumors comprise a group of heterogeneous lesions ranging from hamartomatous or nonneoplastic tissue proliferations to benign neoplasms to malignant tumors with metastatic potential [9]. The odontogenic cysts, which may be intimately associated with the development of certain of the odontogenic tumors, also represent an aberration at some stage of odontogenesis [10]. Keratocystic odontogenic tumor (KCOT) has been recently classified by World Health Organization (WHO) in 2005 as a benign tumor of odontogenic epithelium to emphasize its neoplastic nature [9].

Unicystic ameloblastoma is considered at best an in situ or superficially invasive form of ameloblastoma [11]. Unicystic ameloblastomas are well known to be lined by a variable epithelium ranging from one that has typical ameloblastic characteristics to one that is metaplastic and which appears completely nondescript consisting of several layers of nonkeratinizing squamous cells. In such cases, the differentiation of odontogenic cysts from the unicystic ameloblastoma can be problematic [12].

The histologic presentation of ameloblastoma, especially unicystic type, can be, in some instances, mistaken for keratocystic odontogenic tumor (KCOT). Overlapping clinical and radiographic presentation further adds to this diagnostic difficulty [3].

Hence, the present study was carried out to evaluate and intercompare the expression of calretinin in ameloblastoma and keratocystic odontogenic tumor in order to assess its usefulness as a confirmatory marker for ameloblastic tissue that would aid in the differential diagnosis of such lesions.

## 2. Materials and Methods

### 2.1. Case Selection

Formalin-fixed paraffin-embedded tissue blocks of ameloblastoma (unicystic and multicystic variants) and KCOT (parakeratinized type) were retrieved from the archives of Department of Oral and Maxillofacial Pathology, Pacific Dental College and Hospital, Udaipur. Re-evaluation of all cases was performed according to the World Health Organization histological typing of odontogenic tumors [9] by two oral pathologists on light microscopy using hematoxylin and eosin sections. The study protocol was reviewed by the Ethical Committee of Pacific Dental College and Hospital and was granted ethical clearance.

### 2.2. Immunohistochemistry

Formalin-fixed paraffin-embedded tissue blocks were cut into 4 *μ*m thick sections and placed on organosilane-pretreated slides. Immunohistochemical staining was performed using the primary antibody for calretinin using supersensitive one-step polymer-HRP technique (Biogenex Life Sciences, San Ramon, CA, USA). The sections were deparaffinized and rehydrated through xylene and descending grades of alcohol, respectively. Antigen retrieval was carried out using commercial microwave antigen retrieval system where the sections were immersed in 10 mM sodium citrate buffer (pH 6.0) at 800, 420, and 320°F for three cycles of 10 min each (EZ-Retriever System, Biogenex Life Sciences). After rinsing in tris buffer (pH 7.2), the sections were incubated with 3% hydrogen peroxide in water for 15 min to block the endogenous peroxidase activity. This was followed by a power block for 10 min at room temperature to block any nonspecific antigenic sites. The sections were then incubated with optimally prediluted rabbit polyclonal antibody against calretinin protein (Biogenex, CA, USA) for 30 min at room temperature in a moist chamber. After washing with tris buffer (pH 7.2) for 5 min, the sections were then incubated with super enhancer for 20 min at room temperature, followed by secondary antibody incubation with one-step polymer-HRP reagent for 30 min at room temperature in a moist chamber. Visualization was performed using freshly prepared 3,3′-diaminobenzidine hydrochloride (DAB) substrate (Sigma) for 5 min. The slides were counterstained with Mayer's hematoxylin for 3 min, subsequent to which sections were dehydrated, cleared, and mounted with dibutyl phthalate xylene (DPX) (Rankem, RFCL Ltd.). For each batch of staining, positive and negative controls were run simultaneously with the study specimens. Human mesothelioma which is known to have a high level of calretinin expression served as the positive control, while the primary antibodies were replaced by nonimmune mouse serum at the same dilutions for the negative controls.

### 2.3. Evaluation of Slides

The evaluation of the stained slides was carried out using a binocular research microscope (Lawrence & Mayo) under 100x and 400x magnification. The sections stained with calretinin antibody were evaluated for the presence, localization, distribution, and intensity of the immunoreactive cells [4].* Presence* was evaluated to estimate whether the staining was positive or negative and, if positive, which epithelial layer was stained.* Localization *was evaluated to estimate whether the staining was nuclear (N), cytoplasmic (C), or both.* Distribution *was evaluated as being either focal (involving <50% of positive cells) or diffuse (involving >50% of positive cells). The* intensity* was graded based on the number of positive cells seen: 0 = no staining; 1 = weak staining; 2 = moderate staining; 3 = intense staining.

The Chi-square test was applied to intercompare the calretinin expression between ameloblastoma and KCOT and for the comparison between the variants of ameloblastoma (unicystic and multicystic).* P* values <0.05 were considered to be statistically significant.

## 3. Results

A total of 40 cases (20 each) of ameloblastoma and KCOT were studied. Out of the 20 cases of ameloblastoma, 16 were of unicystic type and the remaining 4 were of solid/multicystic type [2 follicular, 1 plexiform, and 1 granular cell ameloblastoma].

### 3.1. Unicystic Ameloblastoma

**Figure 1:**
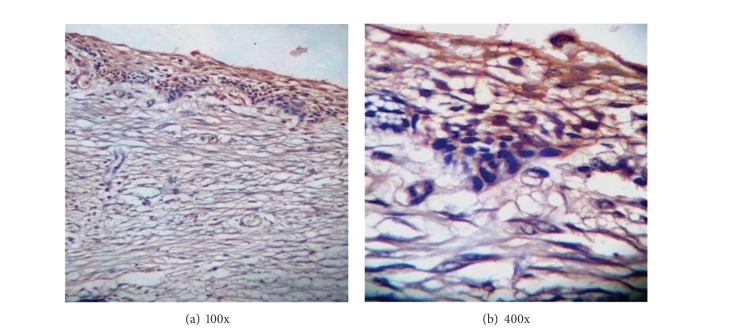
Photomicrograph showing calretinin staining in unicystic ameloblastoma in (a) 100x and (b) 400x.

Out of the 16 cases of unicystic ameloblastoma, positive calretinin expression of the ameloblastic epithelium was seen in 8 (50%) cases. In all the immunopositive cases, reactivity was observed in both the nucleus and cytoplasm of the cells. The distribution of calretinin was focal in 5 and diffuse in 3 cases and was limited to the stellate reticulum like area. The luminal layer of ameloblast-like cells did not stain, except for the few single basal cells that were positive in 2 cases only. Four cases showed weak staining, 3 showed moderate, and 1 showed severe staining of the ameloblastic epithelium (Figure 1).

### 3.2. Solid/Multicystic Ameloblastoma

**Figure 2:**
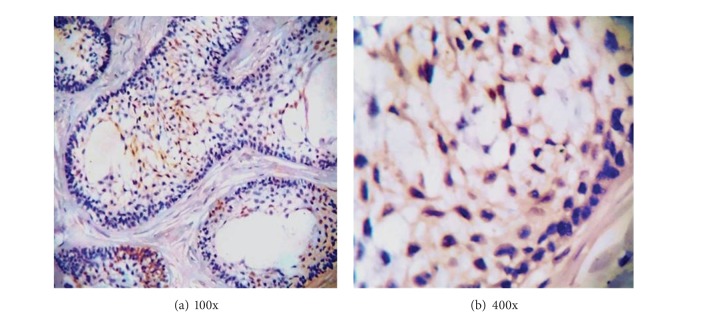
Photomicrograph showing calretinin staining in solid multicystic ameloblastoma in (a) 100x and (b) 400x.

All the 4 cases showed weak positive staining of stellate reticulum like areas whereas only one case showed focal positivity in the basal cell layer also. Immunoreactivity was observed in both the nucleus and cytoplasm of the cells. Staining was focally distributed in 3 cases while one case stained diffusely (Figure 2).

In addition, all the cases of ameloblastoma also showed scattered positive cells within the stroma, which were thought to be nonepithelial in type and could represent mast cells.

### 3.3. Keratocystic Odontogenic Tumor

**Figure 3:**
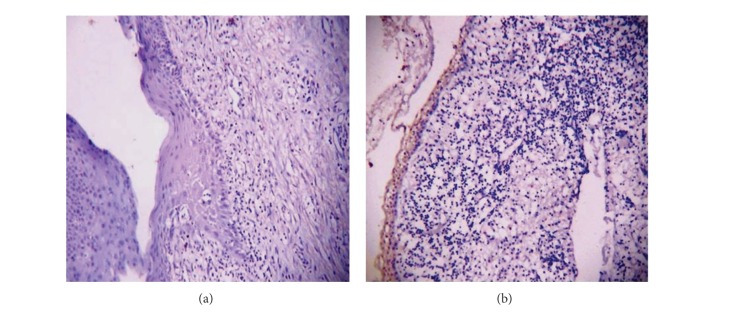
Photomicrograph showing (a) negative calretinin staining in keratocystic odontogenic tumor (100x) and (b) positive calretinin staining in keratocystic odontogenic tumor (100x).

Nineteen (95%) cases showed negative staining of calretinin in the epithelial lining of the cyst, except for some scattered, darkly stained individual cells in the fibrous connective tissue wall. Only one case that showed positivity to calretinin revealed a highly inflamed odontogenic keratocyst with one bit of tissue depicting stellate reticulum like epithelium overlying the palisaded basal cell layer in the hematoxylin and eosin stained sections. The calretinin staining for the same bit showed uniform diffuse immunopositivity of the nucleus and cytoplasm of the stellate reticulum like epithelium (Figure 3).

The intercomparison of calretinin positivity between ameloblastoma and KCOT revealed a statistically significant difference at *P* = 0.000 (Table 1).

## 4. Discussion

Odontogenic cysts and tumors are a group of lesions arising from the tooth-producing apparatus or its remnants. They may originate from odontogenic epithelium and/or ectomesenchyme with varying degrees of inductive tissue interaction [13]. They are rare and lack of familiarity with these lesions and their variable appearance may lead to difficulties in diagnosis with occasional serious confusion with more sinister lesions [14].

Different odontogenic cysts and tumors have variable clinical and biological behaviors. Ameloblastoma is a benign, locally aggressive epithelial odontogenic tumor that has the potential to become malignant and produce metastasis to distant sites such as lungs and kidneys. KCOT is an aggressive cyst with neoplastic behaviour while unicystic ameloblastoma is a neoplasm with cyst like behaviour [15, 16]. KCOT also arises from cell rests of the dental lamina, same origin as ameloblastoma, and there are clinical and radiographic similarities between KCOT and unicystic ameloblastoma, as both present as ordinary cysts in the dentate areas [17]. Histologically, unicystic ameloblastoma is lined in some areas, but rarely entirely, by odontogenic epithelium of ameloblastoma appearance and stratified squamous epithelium in the remaining areas [18]. In fact, such squamous metaplasia is a relatively frequent phenomenon in unicystic ameloblastoma and many of these lesions are lined by such nondescript epithelium, which can create diagnostic confusion with odontogenic cysts [12].

KCOT is characterized, histologically, by a palisaded basal cell layer of basophilic columnar cells and a surface of corrugated parakeratin, sometimes with spongiosis, resembling closely the stellate like reticulum and the acanthomatous differentiation of ameloblastoma. If the tissue sample is small and if the neoplastic epithelium displays reactive changes induced by inflammation, it can closely resemble unicystic ameloblastoma histologically [3]. Thus, at times, both lesions become histologically indistinguishable.

Many techniques have been used in an attempt to distinguish odontogenic cysts (including KCOT) from ameloblastomas (especially unicystic type), which include demonstration of cell surface carbohydrates with blood group specificity; determination of alkaline phosphatase activity in the stroma; distribution of lectins and involucrin in the epithelium; characterization of cytokeratin profiles; counting of AgNORs and quantification of cell proliferation markers such as PCNA and Ki67. While differences have been shown to occur between various cysts and ameloblastomas, considerable overlap exists and none of the above techniques can be used to routinely distinguish these lesions from one another [5].

Also, a number of studies have reported the immunohistochemical expression of intermediate filaments, growth factors, basement membrane components, cell cycle regulating factors, and apoptotic proteins in ameloblastomas but calcium-binding proteins have not been investigated to a greater extent in these tumors [19].

The present study assessed the expression of calretinin in 20 cases each of ameloblastoma and keratocystic odontogenic tumor and the results demonstrated frequent expression of calretinin in the epithelium of both unicystic and solid multicystic ameloblastomas. Positive staining was seen in 50% (8 cases) of unicystic ameloblastoma and 100% (4 cases) of multicystic ameloblastoma, whereas only 1 out of 20 cases of KCOT lining showed positive staining for calretinin. The intercomparison between both groups revealed a statistically significant difference at *P* = 0.000. Also, the immunopositivity was seen exclusively in the stellate reticulum like epithelium in both the unicystic and multicystic ameloblastomas, and the ameloblast-like basal cells were positive in only 1 of the multicystic and 2 of the unicystic variants, where single cell expressing calretinin was observed.

These findings were in accordance with earlier studies which also observed calretinin positive cells restricted to the neoplastic stellate reticulum like epithelium in ameloblastoma only; Altini et al. [19] and Coleman et al. [12] found positive staining in 81.5% cases of unicystic ameloblastomas and 93.5% cases of multicystic ameloblastoma, while Devilliers et al. [3], Sundaragiri et al. [5], and D'Silva et al. [20] found positivity in all cases of ameloblastoma (100%). In all these studies, none of the odontogenic cysts' lining showed positivity, except D'Silva et al. [20] who observed positive staining of the cystic lining epithelium and keratin flakes in the cystic lumen of 40% cases of odontogenic keratocyst. The authors attribute this finding to the aggressive biologic behavior of odontogenic keratocyst similar to a benign neoplasm and its high mitotic activity as compared to other nonneoplastic odontogenic cysts. Also, Piattelli et al. [21] found positivity to calretinin in 8 of 12 parakeratinized keratocysts in the parabasal-intermediate layers of the cyst epithelium. This could point to the aggressive behavior of KCOT and help to explain the differences in the clinical and pathologic behavior of odontogenic keratocysts, in particular the differences found between orthokeratinized keratocysts and parakeratinized keratocysts.

Alaeddini et al. [4] also found that calretinin immunoreactivity was positive only for ameloblastoma when compared to calcifying epithelial odontogenic tumour, adenomatoid odontogenic tumour, ameloblastic fibroma, and odontogenic myxoma, stating that this protein may have a role in the transition of the dental lamina remnants to ameloblastoma. They hypothesized that calretinin may be one of the factors responsible for the differences between this aggressive neoplasm and other odontogenic tumors studied.

Further, in the present study, the distribution pattern of the immunopositive cells was focal in 8 cases (40%) and diffuse in 4 cases (20%). Also, the staining was weak in most of the cases (40%), with 3 cases (15%) showing moderate and only 1 case (5%) intense staining. The staining was not equally distributed throughout the sections with some areas showing intense staining but absolutely no staining in the immediate vicinity. This was in contrast to the earlier studies where most of the cases showed intense staining of the ameloblastic epithelium [3–5, 12, 19, 20].

Altini et al. [19] stated that the better the differentiation of the epithelium was, the lesser the expression of calretinin occurred in their study, where they found little or no immunostaining in those cases of unicystic ameloblastomas that were lined by typical ameloblastic epithelium, while the epithelium which completely lacked ameloblastic features frequently expressed calretinin. Hence they indicated that calretinin expression in some cells varied according to their metabolic activity and may be lost when this activity changes. Later, Coleman et al. [12] observed intense positive staining in both areas of nondescript epithelial lining and areas with typical ameloblastic features in unicystic ameloblastoma, which indicated that although the metaplastic cyst linings may have lost their typical ameloblastic features, the cells have retained their immunophenotypic characteristics resulting in the continued expression of calretinin. Our study can be correlated with the observation of Altini et al. [19] as majority of the unicystic ameloblastoma cases that we observed were lined by typical ameloblastic epithelium, due to which they may not have stained intensely with calretinin.

In a study by Mistry et al. [2] on developing rat molars, calretinin immunoreactivity was present in the inner enamel epithelium and presecretory ameloblasts from the late cap stage onwards. In the cap and late cap stages, many of the specimens were immunopositive for calretinin in the stellate reticulum, and this number increased to >90% for the early and late bell stages. In the present study, the pattern of reactivity of the stellate reticulum observed appeared similar to the late bell stage of normal tooth development. However, in the peripheral layers of the ameloblastic islands, calretinin immunoreactivity was not observed as was the case in normal tooth germs. As enamel organ has been proposed to be one of the possible origins of ameloblastoma [17], this peculiar distribution of calretinin in ameloblastic epithelium is noteworthy and it thus appears that there is no obvious correlation between the staining of normal odontogenic tissues and their neoplastic counterparts [19].

A possible explanation for this dynamic spatial and temporal distribution was that calretinin may be present only in cells that reside directly in the path of calcium in transition on its way to the enamel matrix, acting as a “calcium ferry” [2]. However, Hubbard et al. [22] found that calretinin was primarily expressed during the differentiation stage of enamel formation, and only a faint calretinin band was observed at secretion stage while no such band was detectable during maturation. Based on these findings, the authors suggest retirement of the calcium ferry dogma from enamel biology—in murine models at least.

Gotzos et al. [23, 24] have shown increased expression of calretinin in rapidly proliferating WiDr colon adenocarcinomas cells. Upon being induced to differentiate by treatment with sodium butyrate and hexamethylene bisacetamide, they showed marked reductions in cell proliferation as well as expression of full length calretinin. When treated with oligonucleotides against calretinin synthesis, these cells were blocked in the G1 phase of the cell cycle and subsequently underwent apoptosis. These authors suggested that calretinin may maintain an undifferentiated proliferative state characteristic of neoplastic transformation. Also, calretinin may act as an antiapoptotic factor [25].

The single case of KCOT in our study that showed positivity to calretinin was of a 17-year-old male patient having radiolucency in 24 and 25 region with impacted 23. The hematoxylin and eosin stained sections of the biopsy specimen revealed a highly inflamed odontogenic keratocyst with one bit of tissue depicting stellate reticulum—like epithelium overlying the palisaded basal cell layer. Upon calretinin staining, uniform diffuse immunopositivity of the stellate reticulum like epithelium was observed. This suggests that the previous biopsy may have been misdiagnosed as KCOT on the basis of hematoxylin and eosin staining.

This result can be compared with that of Devilliers et al. [3], wherein they evaluated a tumor from their archives, originally diagnosed as KCOT, which recurred 2 years later. Histologic sections from the tissue biopsied earlier (diagnosed as KCOT) and from the tissue sample of the recurrence (diagnosed as ameloblastoma) were tested with calretinin and both showed positive staining of the neoplastic epithelium, confined to the stellate reticulum.

This finding helps us to understand the importance of calretinin as a differential diagnostic marker for ameloblastoma and how important it is for the pathologist to differentiate both entities, which carry different treatment protocols with potentially serious functional and esthetic consequences for the patient.

Few nonepithelial cells that stained positive for calretinin were also observed in the connective tissue stroma of virtually all cases of ameloblastoma and KCOT in our study. Altini et al. [19] and Coleman et al. [12] also found such darkly stained cells within the tumor or cyst epithelium and in the fibrous connective tissue walls. These were interpreted as being mast cells or Langerhans cells, both of which have been documented as occurring in ameloblastomas and odontogenic cysts.

## 5. Conclusion

The present study suggests that calretinin may be used as a specific immunohistochemical marker for neoplastic ameloblastic epithelium as calretinin positivity was observed exclusively in ameloblastomas. Hence, it can serve as an important diagnostic adjunct in the differential diagnosis of ameloblastoma and keratocystic odontogenic tumor. The expression of calretinin in these neoplastic lesions might be recapitulating dental ontogeny, as its role during normal tooth development has also been speculated.

## Figures and Tables

**Table 1:** Intercomparison of calretinin presence in ameloblastoma and KCOT.

Presence	Ameloblastoma (*n* = 20)		KCOT (*n* = 20)	
	Frequency	Percent	Frequency	Percent
+	12	30%	1	2.5%
−	8	20%	19	47.5%
P* value	0.000			

*P value < 0.05: statistically significant.
